# Controlling Nutritional Status (CONUT) Predicts Survival in Gastric Cancer Patients With Immune Checkpoint Inhibitor (PD-1/PD-L1) Outcomes

**DOI:** 10.3389/fphar.2022.836958

**Published:** 2022-03-04

**Authors:** Li Chen, Hao Sun, Ruihu Zhao, Rong Huang, Hongming Pan, Yanjiao Zuo, Lele Zhang, Yingwei Xue, Hongjiang Song, Xingrui Li

**Affiliations:** ^1^ Department of Thyroid and Breast Surgery, Tongji Hospital, Tongji Medical College of Huazhong University of Science and Technology, Wuhan, China; ^2^ Department of Gastrointestinal Surgery, Harbin Medical University Cancer Hospital, Harbin Medical University, Harbin, China

**Keywords:** gastric cancer, controlling nutritional status, immune checkpoint inhibitors, PD-1, PD-L1

## Abstract

**Objective:** The controlling nutritional status (CONUT), based on total lymphocyte count (TL), total cholesterol level (T-CHOL), and serum albumin (ALB), can provide a useful immunological prognostic biomarker for cancer patients. The present study aims to investigate the correlation between CONUT and prognosis in gastric cancer patients receiving immune checkpoint inhibitor (ICI) treatment.

**Methods:** We retrospectively enrolled 146 patients with gastric cancer treated with ICIs (PD-1/PD-L1 inhibitors) from August 2016 to December 2020. The clinicopathologic characteristics were analyzed by Chi-square test or Fisher’s exact test. The Kaplan–Meier and log-rank test were used to calculate and compare progression-free survival (PFS) and overall survival (OS). The prognostic and predictive factors of PFS and OS were identified by univariate and multivariate analyses. A nomogram was developed to estimate 1-, 3-, and 5-year PFS and OS probability.

**Results:** Through the CONUT score, there were 75 (51.37%) patients in the low CONUT group and 71 (48.63%) patients in the high CONUT group. There was a correlation between the CONUT score and age (*p* = 0.005), pathology (*p* = 0.043), ALB (*p* = 0.020), PALB (*p* = 0.032), and Hb (*p* = 0.001). The CA724, TNM stage, and treatment (ICIs vs. chemotherapy) were the independent prognostic factors for PFS and OS by multivariate analyses. Patients with high CONUT score had poorer PFS and OS (χ^2^ = 3.238, *p* = 0.072, and χ^2^ = 4.298, *p* = 0.038). In the subgroup analysis, the patients with high CONUT score were associated with shorter PFS and OS with ICIs or chemotherapy. With the PD-1/PD-L1 positive expression, the patients with high CONUT score had shorter PFS and OS than those with low CONUT score. Furthermore, the patients with high CA724 value were associated with shorter PFS and OS. The toxicity assessment in ICIs or chemotherapy was significantly associated with anemia. The nomograms were constructed to predict the probability of 1-, 3-, and 5-year PFS, and 1-, 3-, and 5-year OS with C-indices of 0.749 and 0.769, respectively.

**Conclusion:** The CONUT, as a novel immuno-nutritional biomarker, may be useful in identifying gastric cancer patients who are unlikely to benefit from ICI treatment.

## Introduction

Gastric cancer is one of the most commonly diagnosed digestive tract cancers all over the world (Sung et al., 2021). At the present time, radical gastrectomy is the main treatment for gastric cancer, and its clinical course remains unsatisfactory as a result of a considerable number of patients who develop local recurrence or distal metastasis after resection (Joharatnam-Hogan et al., 2020). Although the incidence rate of gastric cancer has gradually declined in recent decades, the total number of patients diagnosed with gastric cancer has been rising, especially in Korea, Japan, Mongolia, and China (Ito et al., 2021). Nowadays, the multidisciplinary treatment, including surgery, perioperative chemotherapy, radiotherapy, and targeted therapy are the main choices for the treatment of gastric cancer (Wagner et al., 2020). Choosing the optimal treatment is very important to improve the patient’s prognosis. Moreover, it is also vital to identify potential biomarkers to select appropriate treatment strategies and predict the prognosis of gastric cancer patients.

The nutritional status is an important factor because it can predict treatment tolerance and cancer progression (Mantzorou et al., 2017). Malnutrition is common in gastric cancer patients due to a decrease in food intake and energy consumption (Loman et al., 2019). The poor nutritional condition is also related to tumor invasion, metabolism, immune impairment, intolerance to cancer treatment, and postoperative complications (Lakananurak and Gramlich, 2020; Abe et al., 2021; Rovesti et al., 2021). Moreover, cachexia, as a complex and multifactorial syndrome, affects about 50%–80% of cancer patients and is related to 20%–40% of cancer deaths (Freire et al., 2019). Multiple nutritional assessment systems, including Naples Prognostic Score (NPS), Nutritional Risk Screening (NRS), body mass index (BMI), albumin (ALB), and prognostic nutritional index (PNI), have emerged with the aim of detecting and predicting the clinical outcomes of gastric cancer patients (Li et al., 2019; Wu et al., 2019; Bae, 2020; Park et al., 2020; Xiong et al., 2020). It is also reported that immunological status is associated with the patient’s prognosis (Sato et al., 2020). There is an indisputable link between nutritional status, systemic inflammation, and carcinogenesis (Alwarawrah et al., 2018). Some inflammatory indicators are monitored routinely before surgery, such as neutrophils, monocytes, lymphocytes, platelet, and C-reactive protein (Wang et al., 2017; Eljaszewicz et al., 2018; Saito et al., 2018; Migita et al., 2019; Mansuri et al., 2021). Furthermore, several reliable combined scoring systems are developed to accurately evaluate the prognosis of patients, for instance, platelet-to-lymphocyte ratio (PLR), neutrophil-to-lymphocyte ratio (NLR), and lymphocyte-to-monocyte ratio (LMR) (Ma and Liu, 2018; Miyamoto et al., 2018; Cao et al., 2020).

The Controlling Nutritional Status (CONUT) score initially reported in 2005 by Ignacio de Ulíbarri J and others as an automatic tool for the early detection and continuous monitoring of malnutrition, and covering laboratory information, including total lymphocyte count, total cholesterol level, and serum albumin (Ignacio de Ulíbarri et al., 2005). In recent years, clinicians and researchers have increased interest in the CONUT score, and several studies have shown that the CONUT score was a validated and useful nutritional status assessment in predicting the prognosis of different types of cancer outcomes (Ahiko et al., 2019; Takagi et al., 2020; Kheirouri and Alizadeh, 2021). Moreover, the effect of CONUT score on the prognosis of patients with gastric cancer has been first reported in 2018 by Kuroda et al. (2018). To date, the prognostic and predictive value of novel inflammatory biomarkers for immune checkpoint inhibitors (ICIs) is unknown in most tumor types. In the present study, we aim to investigate the correlation between CONUT and prognosis in gastric cancer patients who received ICI (PD-1/PD-L1 inhibitor) treatment.

## Materials and methods

### Patients

We respectively analyzed 146 patients with gastric cancer treated with ICIs at the Harbin Medical University Cancer Hospital between August 2016 and December 2020. The clinical data were collected and searched by electronic medical records. All patients’ data that were accessed complied with relevant data protection and privacy regulations. All personal data were handled in strict compliance with the ethical guidelines stipulated by the 1964 Declaration of Helsinki (including its later amendments or similar ethical frameworks). Institutional review board approval was acquired to review medical records at Harbin Medical University Cancer Hospital.

Inclusion criteria were (1) patients pathologically diagnosed with gastric cancer, (2) patients who underwent ICIs or/plus chemotherapy, and (3) Eastern Cooperative Oncology Group (ECOG) score: 0–2. Exclusion criteria were (1) autoimmune disease or systemic immunosuppression, (2) have incomplete clinicopathological data, especially in blood test results, and (3) absence of efficacy assessment.

### Controlling Nutritional Status score

The CONUT score, which comprise three factors, was based on total lymphocyte count (TL), total cholesterol level (T-CHOL), and serum albumin (ALB) in each patient. Information on complete blood cell counts with differential counts within 7 days before treatment was extracted. (1) TL ≥1.60, 1.20–1.59, 0.80–1.19, <0.80 × 10^9^/L were scored as 0, 1, 2, and 3 points, respectively. (2) ALB ≥35.0, 30.0–34.9, 25.0–29.9, <25.0 g/L were scored as 0, 2, 4, and 6 points, respectively. (3) T-CHOL ≥180, 140–179, 100–139, <100 mg/dl were scored as 0, 1, 2, and 3 points, respectively. The CONUT score was defined as the sum of (1) TL, (2) ALB, and (3) T-CHOL (Table 1). In this study, patients were divided into two groups: the low CONUT group (score = 0) (N = 75) and the high CONUT group (score >0) (N = 71).

**TABLE 1:** Definition of Controlling Nutritional Status (CONUT) score.

Parameter	Malnutritional state			
	None	Light	Moderate	Severe
ALB (g/L)	≥35.0	30.0–34.9	25.0–29.9	<25.0
Score	0	2	4	6
TL (×10^9^/L)	≥1.60	1.20–1.59	0.80–1.19	<0.80
Score	0	1	2	3
T-CHOL (mg/dl)	≥180	140–179	100–139	<100
Score	0	1	2	3
Total CONUT score	0–1	2–4	5–8	9–12

Note ^#^ALB, serum albumin; TL, total lymphocyte count; T-CHOL, total cholesterol level, Total CONUT score = ALB score + TL score + T-CHOL score.

### Follow Up

All enrolled patients were routinely followed up by telephone. The date of last follow-up in the present study was November 2021. Progression-free survival (PFS) was defined as the first day of regimen, first day of immunotherapy, or diagnosed with gastric cancer to the date of documented disease progression, death, or last follow-up, respectively. Overall survival (OS) was defined as the first day of regimen, first day of immunotherapy, or diagnosed with gastric cancer to the date of death from any cause or last follow-up, respectively.

### Statistical analysis

Statistical analysis data were statistically analyzed using the R (version 3.6.0; Vienna, Austria. URL: http://www.R-projec t. org/), SPSS software (version 17.0; SPSS Inc., Chicago, IL, USA), and GraphPad Prism software (version 8.0; GraphPad Inc., La Jolla, CA, USA). The qualitative variables were as numbers (percentages) and compared using the Chi-square test or Fisher’s exact test. The quantitative variables were compared using Student’s *t*-test. Survival rate was calculated by Kaplan–Meier survival curve, and the differences were evaluated by log-rank test. The univariate and multivariate Cox proportional hazard regression model was used to evaluate the independent prognostic factors. Hazard ratio (HR) with its 95% confidence interval (CI) was estimated by the univariate and multivariate Cox proportional hazard regression model. Prognostic nomograms for PFS and OS were established on the basis of the multivariate analyses. All *p*-values were two sided, and statistical differences were termed as *p*-value <0.05.

## Results

### Patient Characteristics

Through the CONUT score, 75 (51.37%) patients and 71 (48.63%) patients were allocated to the low CONUT group and high CONUT group, respectively. Details of the enrolled patients’ clinical characteristics are summarized in Table 2. The present study enrolled 146 patients, including 102 (69.9%) men and 44 (30.1%) women. The median age was 59 years and ranged 34–82 years. There were 86 patients who received surgery and 60 patients who did not receive surgery. According to the eighth edition of the TNM classification, 24 (16.4%) and 122 (83.6%) gastric cancer patients were classified as stage I + II and III + IV, respectively. The CONUT was associated with age (*p* = 0.005) and pathology (*p* = 0.043).

**TABLE 2:** Patient characteristics.

	Level	Low CONUT	High CONUT	*p*-Value
N		75	71	
Sex (%)	Male	54 (72.0)	48 (67.6)	0.691
	Female	21 (28.0)	23 (32.4)	
Age (median [IQR])		62.0 [54.5, 66.0]	57.0 [48.0, 64.5]	0.010
Age (%)	<59	26 (34.7)	42 (59.2)	0.005
	≥59	49 (65.3)	29 (40.8)	
BMI (%)	<21.55	35 (46.7)	38 (53.5)	0.508
	≥21.55	40 (53.3)	33 (46.5)	
ABO blood type (%)	A + B	41 (54.7)	45 (63.4)	0.367
	O + AB	34 (45.3)	26 (36.6)	
SLNM (%)	No	63 (84.0)	65 (91.5)	0.256
	Yes	12 (16.0)	6 (8.5)	
ECG (%)	Normal	54 (72.0)	50 (70.4)	0.978
	Abnormal	21 (28.0)	21 (29.6)	
Surgery (%)	Yes	45 (60.0)	41 (57.7)	0.914
	No	30 (40.0)	30 (42.3)	
Primary tumor site (%)	Upper 1/3	8 (10.7)	13 (18.3)	0.281
	Middle 1/3	21 (28.0)	24 (33.8)	
	Low 1/3	41 (54.7)	28 (39.4)	
	Whole	5 (6.7)	6 (8.5)	
Borrmann type (%)	Borrmann I + II	4 (5.3)	5 (7.0)	0.849
	Borrmann III + IV	41 (54.7)	36 (50.7)	
	Unknown	30 (40.0)	30 (42.3)	
Tumor size (%)	<50 mm	22 (29.3)	19 (26.8)	0.621
	≥50 mm	14 (18.7)	18 (25.4)	
	Unknown	39 (52.0)	34 (47.9)	
Differentiation (%)	Poor	49 (65.3)	46 (64.8)	0.091
	Moderately + Well	24 (32.0)	17 (23.9)	
	Unknown	2 (2.7)	8 (11.3)	
Pathology (%)	Adenocarcinoma	53 (70.7)	44 (62.0)	0.043
	Others^a^	20 (26.7)	17 (23.9)	
	Unknown	2 (2.7)	10 (14.1)	
TNM stage (%)	I + II	14 (18.7)	10 (14.1)	0.601
	III + IV	61 (81.3)	61 (85.9)	
Lauren type (%)	Intestinal	20 (26.7)	13 (18.3)	0.660
	Diffuse	11 (14.7)	10 (14.1)	
	Mixed	11 (14.7)	12 (16.9)	
	Unknown	33 (44.0)	36 (50.7)	
PD-1 (%)	Negative	34 (45.3)	31 (43.7)	0.863
	Positive	9 (12.0)	7 (9.9)	
	Unknown	32 (42.7)	33 (46.5)	
PD-L1 (%)	Negative	21 (28.0)	21 (29.6)	0.761
	Positive	22 (29.3)	17 (23.9)	
	Unknown	32 (42.7)	33 (46.5)	
Treatment (%)	ICIs	44 (58.7)	45 (63.4)	0.679
	Chemotherapy	31 (41.3)	26 (36.6)	

a Others: mucinous carcinoma, signet ring cell carcinoma, mixed carcinoma, unknown; BMI, body mass index; SLNM, supraclavicular lymph node metastasis; ECG, electrocardiogram.

### Nutritional and Blood Parameters

The median total protein (TP), ALB, globulin (GLOB), A/G, prealbumin (PALB), T-CHOL, triglyceride (TRIG), hemoglobin (Hb), carcinoembryonic antigen (CEA), alpha fetoprotein (AFP), carbohydrate antigen199 (CA199), carbohydrate antigen724 (CA724), carbohydrate antigen125II (CA125II) were 68.70 g/L, 38.95 g/L, 29.10 g/L, 1.3, 200 mg/L, 4.25 mmol/L, 1.08 mmol/L, 122.50 g/L, 2.54 ng/ml, 3.02 ng/ml, 14.40 U/ml, 2.56 U/ml, 27.59 U/ml, respectively. The CONUT was associated with ALB (*p* = 0.020), PALB (*p* = 0.032), and Hb (*p* = 0.001). The detailed information is shown in Table 3.

**TABLE 3:** Nutritional and blood parameters.

	Level	Low CONUT	High CONUT	*p*-Value
n		75	71	
TP (%)	<68.70	31 (41.3)	41 (57.7)	0.069
	≥68.70	44 (58.7)	30 (42.3)	
ALB (%)	<38.95	30 (40.0)	43 (60.6)	0.020
	≥38.95	45 (60.0)	28 (39.4)	
GLOB (%)	<29.10	34 (45.3)	39 (54.9)	0.320
	≥29.10	41 (54.7)	32 (45.1)	
A/G (%)	<1.3	29 (38.7)	25 (35.2)	0.794
	≥1.3	46 (61.3)	46 (64.8)	
PALB (%)	<200	30 (40.0)	42 (59.2)	0.032
	≥200	45 (60.0)	29 (40.8)	
T-CHOL (%)	<4.25	34 (45.3)	36 (50.7)	0.629
	≥4.25	41 (54.7)	35 (49.3)	
TRIG (%)	<1.08	36 (48.0)	36 (50.7)	0.872
	≥1.08	39 (52.0)	35 (49.3)	
Hb (%)	<122.50	27 (36.0)	46 (64.8)	0.001
	≥122.50	48 (64.0)	25 (35.2)	
CEA (%)	<2.54	37 (49.3)	36 (50.7)	1.000
	≥2.54	38 (50.7)	35 (49.3)	
AFP (%)	<3.02	33 (44.0)	40 (56.3)	0.185
	≥3.02	42 (56.0)	31 (43.7)	
CA199 (%)	<14.40	39 (52.0)	33 (46.5)	0.616
	≥14.40	36 (48.0)	38 (53.5)	
CA724 (%)	<2.56	39 (52.0)	34 (47.9)	0.741
	≥2.56	36 (48.0)	37 (52.1)	
CA125Ⅱ (%)	<27.59	40 (53.3)	33 (46.5)	0.508
	≥27.59	35 (46.7)	38 (53.5)	

Note. TP, total protein; GLOB, Globulin; PALP, prealbumin; T-CHOL, total cholesterol level; TRIG, triglyceride; Hb, hemoglobin; CEA, carcinoembryonic antigen; AFP, alpha fetoprotein; CA199, carbohydrate antigen199; CA724, carbohydrate antigen724.

### Univariate and Multivariate Cox Hazard Analysis of Biomarkers for Progression-Free Survival and Overall Survival

The univariate analysis indicated that PALB, CEA, CA199, CA724, CONUT, ALB, radical resection, surgery, TNM stage, Lauren type, treatment, PD-1, and PD-L1 were related to the patients’ prognosis for PFS; however, the multivariate analysis showed that CA724, TNM stage, and treatment were the independent prognostic factors for PFS (Table 4). Moreover, the univariate analysis found that PALB, CEA, CA199, CA724, CONUT, ALB, radical resection, surgery, Borrmann type, TNM stage, Lauren type, treatment, PD-1, and PD-L1 were related to the patients’ prognosis for PFS; however, the multivariate analysis showed that CA724, TNM stage, and treatment were the independent prognostic factors for OS (Table 4).

**TABLE 4:** Univariate and multivariate Cox hazard analysis of biomarkers for progression-free survival (PFS) and overall survival (OS).

Parameters		PFS				OS		*p*-Value
	Univariate analysis		Multivariate analysis		Univariate analysis		Multivariate analysis	
	Hazard ratio (95% CI)	*p*-Value	Hazard ratio (95% CI)	*p*-Value	Hazard ratio (95% CI)	*p*-Value	Hazard ratio (95% CI)	
Sex (male vs. female)	1.055 (0.6195–1.798)	0.843			1.084 (0.6361–1.846)	0.768		
Age (<59 vs. ≥59)	0.881 (0.535–1.450)	0.617			0.876 (0.532–1.442)	0.603		
BMI (<21.55 vs. ≥21.55)	0.903 (0.549–1.487)	0.690			0.871 (0.529–1.434)	0.588		
TP (<68.70 vs. ≥68.70)	0.804 (0.488–1.324)	0.391			0.789 (0.479–1.299)	0.351		
GLOB (<29.10 vs. ≥29.10)	1.258 (0.762–2.075)	0.370			1.245 (0.755–2.054)	0.391		
A/G (<1.3 vs. ≥1.3)	0.718 (0.432–1.196)	0.203			0.734 (0.441–1.221)	0.233		
PALB (<200 vs. ≥200)	0.488 (0.292–0.816)	0.006	0.645 (0.353–1.175)	0.152	0.485 (0.291–0.811)	0.006	0.696 (0.371–1.306)	0.259
T-CHOL (<4.25 vs. ≥4.25)	1.014 (0.616–1.669)	0.958			0.988 (0.600–1.626)	0.961		
TRIG (<1.08 vs. ≥1.08)	1.314 (0.796–2.168)	0.286			1.2680 (0.769–2.092)	0.353		
ABO blood type (A + B vs. O + AB)	1.463 (0.889–2.409)	0.135			1.404 (0.853–2.311)	0.182		
Hb (<122.50 vs. ≥122.50)	1.090 (0.662–1.794)	0.735			1.051 (0.639–1.730)	0.844		
CEA (<2.54 vs. ≥2.54)	2.105 (1.255–3.528)	0.005	1.529 (0.828–2.825)	0.175	2.154 (1.282–3.618)	0.004	1.606 (0.857–3.010)	0.139
AFP (<3.02 vs. ≥3.02)	0.822 (0.498–1.355)	0.441			0.833 (0.505–1.373)	0.473		
CA199 (<14.40 vs. ≥14.40)	1.930 (1.155–3.227)	0.012	1.084 (0.619–1.897)	0.778	1.973 (1.181–3.298)	0.009	1.149 (0.651–2.028)	0.631
CA724 (<2.56 vs. ≥2.56)	2.424 (1.430–4.110)	0.001	1.977 (1.090–3.588)	0.025	2.370 (1.398–4.018)	0.001	1.858 (1.010–3.420)	0.047
CA125Ⅱ (<27.59 vs. ≥27.59)	1.124 (0.682–1.851)	0.647			1.168 (0.709–1.924)	0.542		
CONUT (score≤0 vs. >0)	1.688 (1.018–2.798)	0.043	1.121 (0.604–2.081)	0.718	1.697 (1.023–2.813)	0.040	1.041 (0.558–1.943)	0.898
ALB (score≤0 vs. >0)	2.994 (1.653–5.424)	0.000	2.148 (0.897–5.147)	0.086	3.193 (1.759–5.795)	0.000	2.321 (0.963–5.594)	0.061
TL (score≤0 vs. >0)	1.508 (0.9152–2.483)	0.107			1.509 (0.916–2.485)	0.106		
Radical resection (R0 vs. non R0)	2.568 (1.499–4.401)	0.001	1.778 (0.647–4.892)	0.265	2.800 (1.631–4.807)	0.000	1.989 (0.719–5.502)	0.185
Surgery (yes vs. no)	1.942 (1.155–3.263)	0.012	0.778 (0.329–1.842)	0.568	2.093 (1.242–3.526)	0.006	1.350 (0.318–5.724)	0.684
Primary tumor site (low 1/3 vs. others)	0.953 (0.579–1.569)	0.850			0.985 (0.598–1.622)	0.952		
Borrmann type (I + II vs. III + IV + unknown)	1.545 (0.973–2.455)	0.065			1.636 (1.023–2.616)	0.040	0.557 (0.182–1.704)	0.305
Tumor size (<50 mm vs. ≥50 mm + unknown)	1.121 (0.839–1.497)	0.440			1.140 (0.853–1.523)	0.377		
Differentiation (Poor vs. moderate + well + unknown)	0.889 (0.577–1.368)	0.592			0.857 (0.553–1.327)	0.488		
Pathology (adenocarcinoma vs. others)	1.363 (0.936–1.983)	0.106			1.380 (0.947–2.011)	0.094		
TNM stage (I + II vs. III + IV)	6.530 (2.037–20.940)	0.002	6.168 (1.620–23.450)	0.008	6.605 (2.060–21.180)	0.001	7.256 (1.850–28.490)	0.005
Lauren type (intestinal vs. diffuse + mixed + unknown)	1.390 (1.105–1.747)	0.005	1.028 (0.716–1.475)	0.883	1.426 (1.133–1.796)	0.002	1.015 (0.694–1.483)	0.941
Treatment (ICIs vs. chemotherapy)	4.354 (1.354–14.000)	0.014	12.120 (2.270–64.670)	0.003	4.661 (1.448–1.500)	0.010	16.280 (2.930–90.410)	0.001
PD-1 (negative + unknown vs. positive)	1.677 (1.260–2.232)	0.000	0.704 (0.310–1.598)	0.402	1.694 (1.272–2.256)	0.000	0.646 (0.282–1.481)	0.302
PD-L1 (negative + unknown vs. positive)	1.833 (1.313–2.558)	0.000	1.805 (0.700–4.654)	0.221	1.863 (1.336–2.597)	0.000	2.129 (0.819–5.533)	0.121

Note #Primary tumor site: Others, upper 1/3 + Middle 1/3 + whole. Pathology: others, mucinous carcinoma + signet ring cell carcinoma + mixed carcinoma + unknown. CA125II, carbohydrate antigen125II; ICIs, immune checkpoint inhibitors.

### Survival for Serum Albumin, Total Lymphocyte Count, and Controlling Nutritional Status

Through the nutritional assessment system in Table 1, the ALB was divided into two groups: 1) low ALB group: score 0 (ALB≥35.0 g/L), 2) high ALB group: score >0 (ALB<35.0 g/L); and the TL was divided into two groups: 1) low TL group: score 0 (TL ≥1.60 × 10^9^/L), 2) high TL group: score >0 (TL<1.60 × 10^9^/L); and the T-CHOL was divided into two groups: 1) low T-CHOL group: score 0 (T-CHOL≥180 mg/dl), 2) high T-CHOL group: score >0 (T-CHOL<180 mg/dl); and the CONUT was divided into two groups: 1) low CONUT group: score 0, 2) high CONUT group: score >0; respectively. Of all enrolled patients, the score by T-CHOL is 0. Therefore, we further analyzed the survival for ALB, TL, and CONUT.

**FIGURE 1:**
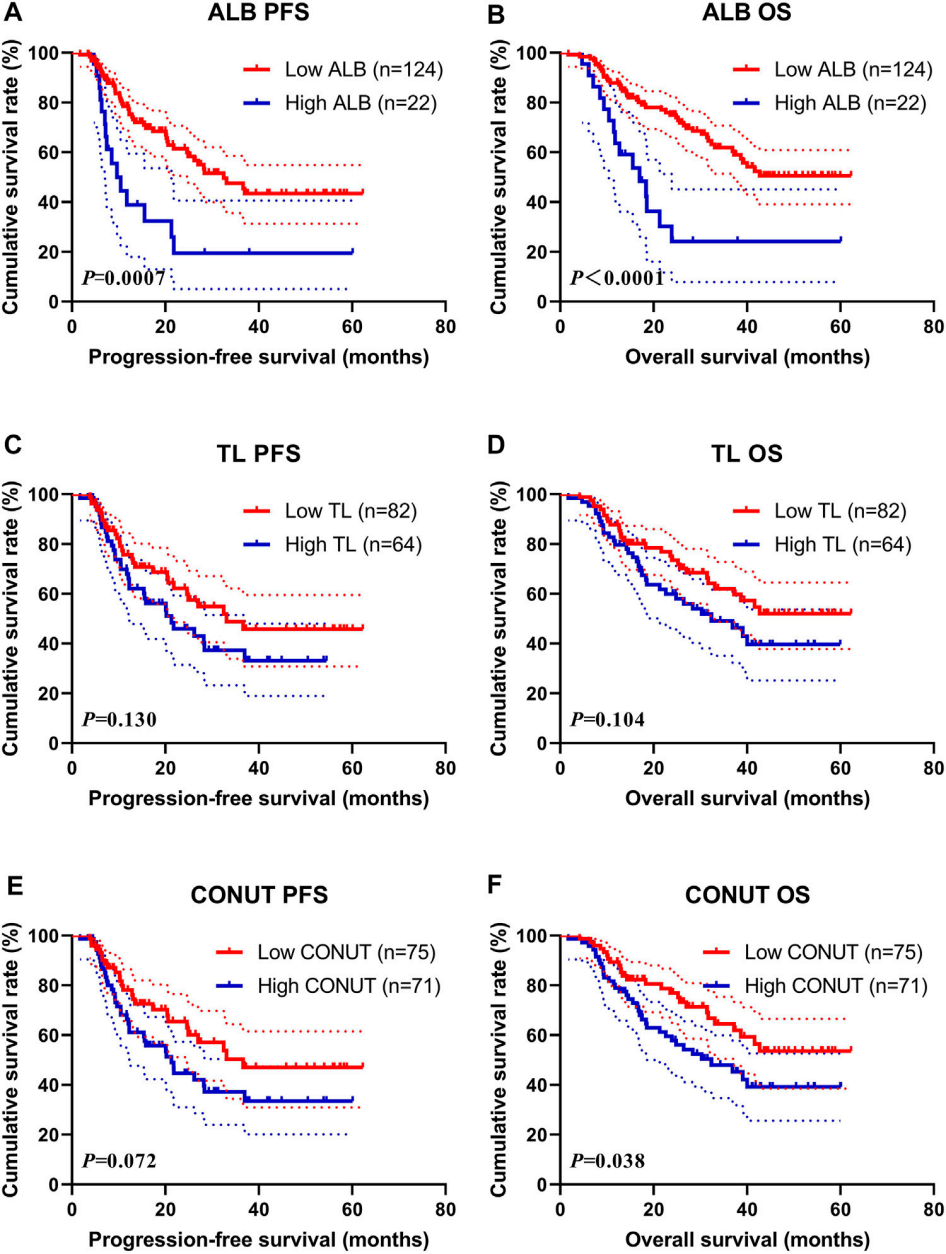
Survival according to the ALB, TL, and CONUT groups for **(A)** progression-free survival (PFS) by ALB; **(B)** overall survival (OS) by ALB; **(C)** PFS by TL; **(D)** OS by TL; **(E)** PFS by CONUT; **(F)** OS by CONUT. ALB, serum albumin; TL, total lymphocyte count; CONUT, Controlling Nutritional Status.

The median PFS and OS in low ALB group (score 0) were 32.50 months vs. not reached, and 9.67 vs. 17.00 months in high ALB group (score >0), respectively. High ALB score was associated with shorter PFS and OS (χ^2^ = 11.410, *p* = 0.0007 and χ^2^ = 16.220, *p* < 0.0001, respectively) (Figures 1A, B). Furthermore, the 1-, 3-, and 5-year survival rates for PFS and OS in low ALB group were 78.5% (95% CI: 71.0%–86.7%), 47.5% (95% CI: 37.2%–60.7%), 43.4% (95% CI: 32.9%–57.1%); and 87.9% (95% CI: 82.3%–93.8%), 61.9% (95% CI: 52.9%–72.4%), 50.6% (95% CI: 40.7%–62.8%), respectively. The 1-, 3-, and 5-year survival rates for PFS and OS in high ALB group were 38.9% (95% CI: 22.1%–68.4%), 19.4% (95% CI: 7.3%–51.8%), 19.4% (95% CI: 7.3%–51.8%), and 63.6% (95% CI: 46.4%–87.3%), 24.2% (95% CI: 10.6%–55.1%), 24.2% (95% CI: 10.6%–55.1%), respectively.

The median PFS and OS in low TL group (score 0) were 33.07 months vs. not reached and 21.33 vs. 32.40 months in the high TL group (score >0), respectively. High TL score was associated with shorter PFS and OS (χ^2^ = 2.291, *p* = 0.130 and χ^2^ = 2.646, *p* = 0.104, respectively) (Figures 1C, D). Furthermore, the 1-, 3-, and 5-year survival rates for PFS and OS in the low TL group were 75.7% (95% CI: 66.2%–86.6%), 48.8% (95% CI: 36.3%–65.5%), 45.8% (95% CI: 33.2%–63.0%); and 87.8% (95% CI: 81.0%–95.2%), 62.1% (95% CI: 51.1%–75.4%), 52.1% (95% CI: 40.1%–67.5%), respectively. The 1- and 3-year survival rates for PFS and OS in the high TL group were 68.0% (95% CI: 56.7%–81.5%), 37.3% (95% CI: 25.3%–55.0%), and 79.7% (95% CI: 70.4%–90.2%), 49.2% (95% CI: 37.4%–64.7%), respectively.

The median PFS and OS in the low CONUT group (score 0) were 36.63 months vs. not reached, and 21.33 vs. 32.40 months in the high CONUT group (score >0), respectively. The high CONUT score was associated with shorter PFS and OS (χ^2^ = 3.238, *p* = 0.072 and χ^2^ = 4.298, *p* = 0.038, respectively) (Figures 1E, F). Furthermore, the 1-, 3-, and 5-year survival rates for PFS and OS in the low CONUT group were 78.1% (95% CI: 68.5%–89.1%), 50.4% (95% CI: 37.2%–68.3%), 47.0% (95% CI: 33.7%–65.6%); and 89.3% (95% CI: 82.6%–96.6%), 64.5% (95% CI: 53.1%–78.3%), and 53.6% (95% CI: 41.1%–69.9%), respectively. The 1-, 3-, and 5-year survival rate for PFS and OS in the high CONUT group were 66.4% (95% CI: 55.6%–79.3%), 37.2% (95% CI: 25.8%–53.5%), 33.5% (95% CI: 22.0%–50.8%); and 78.9% (95% CI: 69.9%–89.0%), 47.9% (95% CI: 36.7%–62.6%), 39.2% (95% CI: 27.6%–55.8%), respectively.

### Treatment (Immune Checkpoint Inhibitors and Chemotherapy)

**FIGURE 2:**
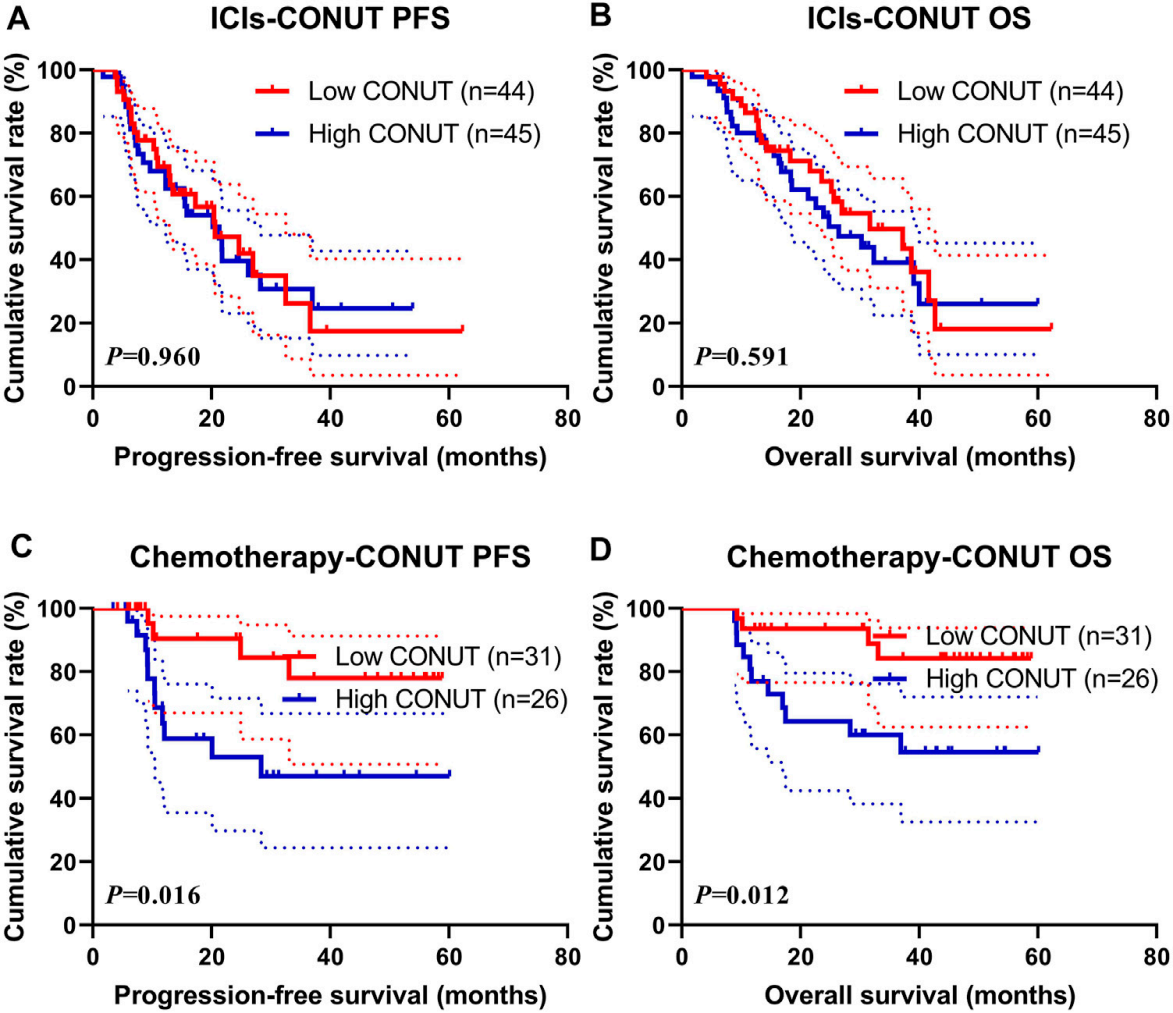
Survival according to the ICIs and Chemotherapy groups for **(A)** progression-free survival (PFS) by ICIs; **(B)** overall survival (OS) by ICIs; **(C)** PFS by Chemotherapy; **(D)** OS by Chemotherapy. ICIs, immune checkpoint inhibitors.

In this study, 89 patients received ICIs treatment (name ICIs group), and 57 patients received chemotherapy (also include targeted therapy or immunotherapy) treatment (name Chemotherapy group). In the ICIs group, the median PFS and OS in the low CONUT group were 20.60 vs. 31.73 months, and 21.33 vs. 26.47 months in the high CONUT group, respectively. The patients with a high CONUT were associated with shorter PFS and OS (χ^2^ = 0.003, *p* = 0.960 and χ^2^ = 0.289, *p* = 0.591, respectively) (Figures 2A, B). Moreover, the 1-, 3-, and 5-year survival rates for PFS and OS in the low CONUT group were 69.4% (95% CI: 56.3%–85.6%), 26.2% (95% CI: 11.7%–58.8%), 17.5% (95% CI: 5.6%–54.5%); and 86.4% (95% CI: 76.8%–97.1%), 49.7% (95% CI: 34.7%–71.0%), 18.1% (95% CI: 5.8%–56.3%), respectively. The 1- and 3-year survival rate for PFS and OS in the high CONUT group were 68.0% (95% CI: 54.9%–84.1%), 30.8% (95% CI: 17.8%–53.3%); and 80.0% (95% CI: 69.1%–92.6%), 39.1% (95% CI: 25.3%–60.4%), respectively.

In the Chemotherapy group, the median PFS and OS in the low CONUT group were not reached vs. not reached, and 28.37 months vs. not reached in the high CONUT group, respectively. The patients with high CONUT were associated with shorter PFS and OS (χ^2^ = 5.764, *p* = 0.016 and χ^2^ = 6.310, *p* = 0.012, respectively) (Figures 2C, D). Moreover, the 1- and 3-year survival rates for PFS and OS in the low CONUT group were 90.5% (95% CI: 78.8%–100.0%), 77.9% (95% CI: 60.7%–100.0%); and 93.5% (95% CI: 85.3%–100.0%), 84.2% (95% CI: 70.8%–100.0%), respectively. The 1-, 3-, and 5-year survival rates for PFS and OS in the high CONUT group were 63.7% (95% CI: 46.4%–87.5%), 47.0% (95% CI: 29.4%–75.3%), 47.0% (95% CI: 29.4%–75.3%), and 76.9% (95% CI: 62.3%–94.9%), 60.0% (95% CI: 43.5%–82.9%), 54.6% (95% CI: 37.6%–79.2%), respectively.

### Surgery (Surgery and Non-Surgery)

**FIGURE 3:**
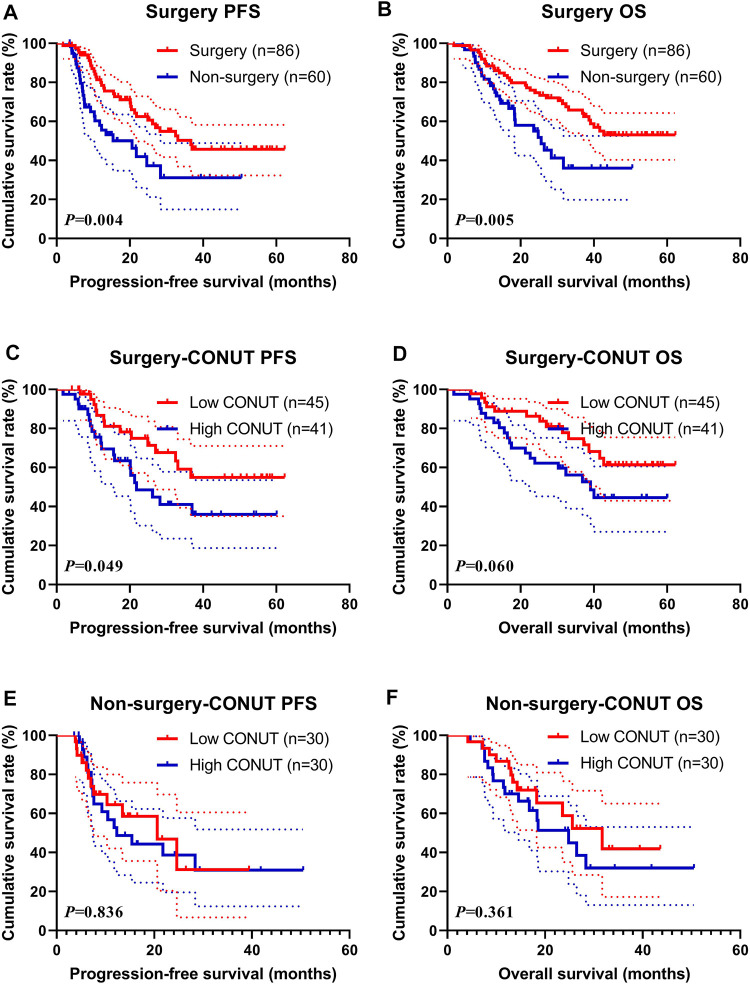
Survival according to the Surgery groups for **(A)** progression-free survival (PFS) by Surgery for all patients; **(B)** overall survival (OS) by Surgery for all patients; **(C)** PFS by Surgery; **(D)** OS by Surgery; **(E)** PFS by Non-surgery; and **(F)** OS by Non-surgery.

In this study, 86 patients received surgery (named Surgery group), and 60 patients did not receive surgery treatment (named Non-surgery group). Between the two groups, the median PFS and OS in the Surgery group were 36.63 vs. not reached, and 20.60 vs. 25.63 months in the Non-surgery group, respectively, and there are statistically significant differences between the two groups (χ^2^ = 8.129, *p* = 0.004 and χ^2^ = 7.998, *p *= 0.005, respectively) (Figures 3A, B). Moreover, the 1-, 3-, and 5-year survival rate for PFS and OS in the Surgery group were 81.4% (95% CI: 73.0%–90.7%), 50.3% (95% CI: 38.9%–65.0%), 45.7% (95% CI: 34.3%–61.0%); and 88.4% (95% CI: 81.9%–95.4%), 65.8% (95% CI: 55.9%–77.4%), 53.0% (95% CI: 42.2%–66.7%), respectively. The 1- and 3-year survival rate for PFS and OS in the Non-surgery group were 58.0% (95% CI: 45.6%–73.8%), 31.1% (95% CI: 17.6%–55.1%); and 78.3% (95% CI: 68.6%–89.5%), 36.0% (95% CI: 22.5%–57.8%), respectively.

In the Surgery group, the median PFS and OS in the low CONUT group were not reached vs. not reached, and 21.80 vs. 39.07 months in the high CONUT group, respectively. The patients with high CONUT were associated with shorter PFS and OS (χ^2^ = 3.853, *p* = 0.049 and χ^2^ = 3.528, *p* = 0.060, respectively) (Figures 3C, D). Moreover, the 1-, 3-, and 5-year survival rates for PFS and OS in the low CONUT group were 86.7% (95% CI: 76.4%–98.3%), 59.2% (95% CI: 43.8%–80.0%), 54.9% (95% CI: 39.3%–76.8%); and 91.1% (95% CI: 83.2%–99.8%), 74.8% (95% CI: 62.1%–89.9%), 61.4% (95% CI: 47.0%–80.2%), respectively. The 1-, 3-, and 5-year survival rate for PFS and OS in the high CONUT group were 75.5% (95% CI: 62.7%–90.9%), 41.1% (95%CI: 26.6–63.3%), 35.9% (95%CI:21.7–59.5%), and 85.4% (95%CI: 75.2–96.9%), 56.1% (95%CI: 42.2–74.5%), 44.5% (95%CI: 30.2%–65.6%), respectively.

In the Non-surgery group, the median PFS and OS in the low CONUT group were 20.60 vs. 31.73 months, and 12.30 vs. 24.83 months in the high CONUT group, respectively. The patients with the high CONUT were associated with shorter PFS and OS (χ^2^ = 0.043, *p* = 0.836 and χ^2^ = 0.836, *p* = 0.361, respectively) (Figures 3E, F). Moreover, the 1- and 3-year survival rate for PFS and OS in the low CONUT group were 64.4% (95% CI: 47.8%–86.8%), 31.2% (95% CI: 11.7%–83.0%); and 86.7% (95% CI: 75.3%–99.7%), 41.9% (95% CI: 22.7%–77.3%), respectively. The 1-, and 3-years survival rates for PFS and OS in the high CONUT group were 52.7% (95% CI: 36.4%–76.4%), 31.0% (95% CI: 15.8%–60.9%); and 70.0% (95% CI: 55.4%–88.5%), 32.1% (95% CI: 16.5%–62.2%), respectively.

### Survival for PD-1/PD-L1 Positive Expression

**FIGURE 4:**
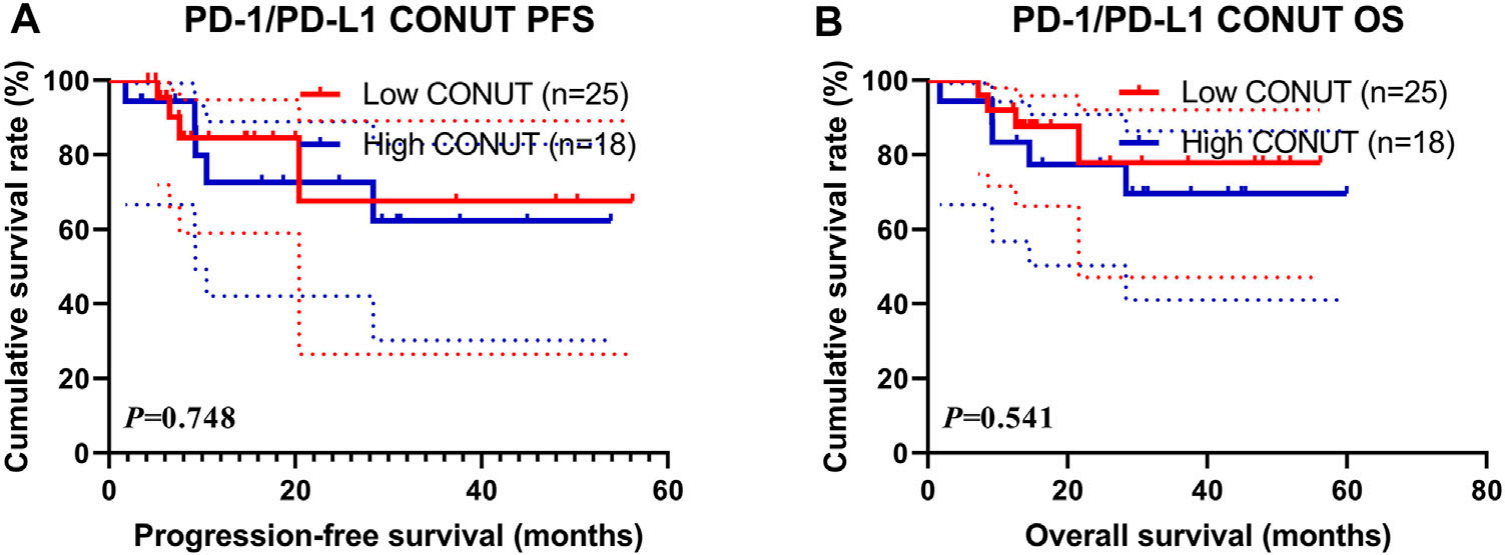
Survival according to PD-1/PD-L1 positive expression groups for **(A)** progression-free survival (PFS); **(B)** overall survival (OS).

In the present study, the PD-1/PD-L1 expression was analyzed on tumor cells by immunohistochemistry (IHC), and the expression of at least 1% was considered positive (Zayac and Almhanna, 2020). Of all enrolled patients, 43 patients (29.5%) had a PD-1/PD-L1 positive expression. Through the CONUT score, 25 (58.14%) patients were in the low CONUT group, and 18 (41.86%) patients were in the high COUNT group, respectively. The patients with the high CONUT score were associated with shorter PFS and OS (χ^2^ = 0.103, *p* = 0.748 and χ^2^ = 0.373, *p* = 0.541, respectively; Figures 4A, B). Moreover, the 1- and 3-year survival rates for PFS and OS in the low CONUT group were 84.5% (95% CI: 69.8%–100.0%), 67.6% (95% CI: 41.9%–100.0%); and 92.0% (95% CI: 82.0%–100.0%), 77.9% (95% CI: 59.1%–100.0%), respectively. The 1- and 3-year survival rates for PFS and OS in the high CONUT group were 72.6% (95% CI: 52.9%–99.9%), 62.3% (95% CI: 40.1%–96.6%); and 83.3% (95% CI: 67.8%–100.0%), 69.6% (95% CI: 50.3%–96.5%), respectively.

### Correlation of the Controlling Nutritional Status Score With Carbohydrate Antigen724

**FIGURE 5:**
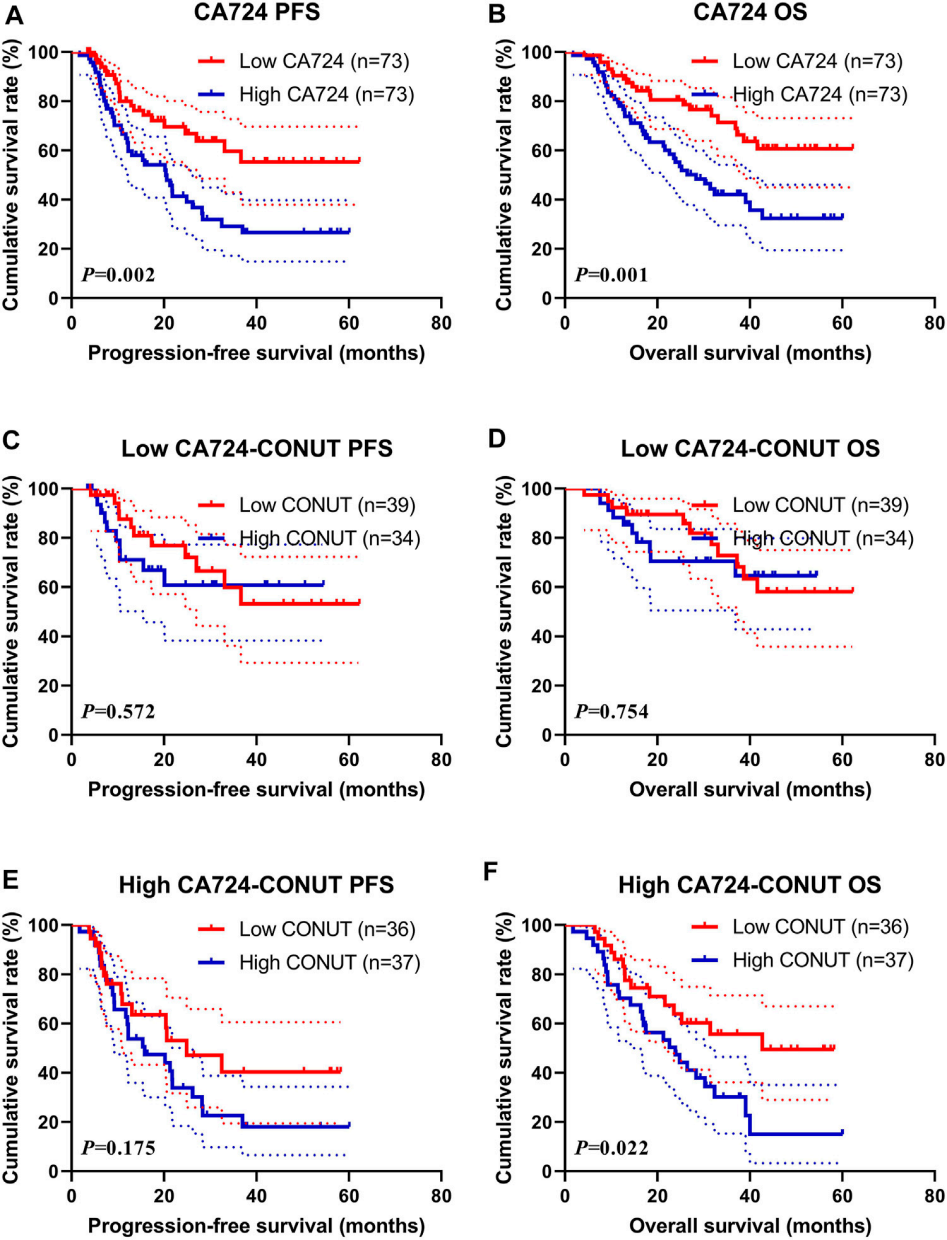
Survival according to carbohydrate antigen 724 (CA724) groups for **(A)** progression-free survival (PFS) by CA724; **(B)** overall survival (OS) by CA724; **(C)** PFS by low CA724; **(D)** OS by low CA724; **(E)** PFS by high CA724; **(F)** OS by high CA724.

Through univariate and multivariate analyses, the CA724 was the significant prognostic factor. To further investigate the prognostic efficiency of CONUT, we analyzed the CA724 by CONUT. The CA724 was divided into two groups by the median value: the low CA724 group and the high CA724 group. Of all enrolled patients, the patients with high CA724 value were associated with shorter PFS and OS (χ^2^ = 9.564, *p* = 0.002 and χ^2^ = 10.900, *p* = 0.001, respectively; Figures 5A, B). Moreover, the 1-, 3-, and 5-year survival rates for PFS and OS in the low CA724 group were 80.0% (95% CI: 70.5%–90.9%%), 59.6% (95% CI: 46.3%–76.8%), 55.4% (95% CI: 41.3%–74.1%); and 90.4% (95% CI: 83.9%–97.4%), 71.5% (95% CI: 60.4%–84.6%), 60.7% (95% CI: 48.1%–76.6%), respectively. The 1-, 3-, and 5-year survival rate for PFS and OS in the high CA724 group were 65.0% (95% CI: 54.3%–77.8%), 29.2% (95% CI: 18.8%–45.5%), 26.6% (95% CI: 16.5%–43.0%); and 78.1% (95% CI: 69.1%–88.2%), 42.2% (95% CI: 31.4%–56.7%), 32.4% (95% CI: 21.3%–49.4%), respectively.

In the subgroup analysis for patients with low CA724 value, patients with high CONUT had shorter PFS and OS than those with low CONUT (χ^2^ = 0.320, *p* = 0.572, and χ^2^ = 0.098, *p* = 0.754, respectively; Figures 5C, D). Moreover, the 1-, 3-, and 5-year survival rate for PFS and OS in the low CONUT group were 87.6% (95% CI: 76.9%–99.8%%), 59.9% (95% CI: 42.2%–85.0%), 53.2% (95% CI: 35.0%–80.9%); and 92.3% (95% CI: 84.3%–100.0%), 72.8% (95% CI: 57.7%–91.8%), 58.1% (95% CI: 41.1%–82.1%), respectively. The 1- and 3-year survival rate for PFS and OS in the high CONUT group were 71.1% (95% CI: 55.9%–90.4%), 60.8% (95% CI: 43.8%–84.3%); and 88.2% (95% CI: 78.0%–99.8%), 70.5% (95% CI: 55.8–89.1%), respectively.

Moreover, in the subgroup analysis for patients with high CA724 value, patients with high CONUT had shorter PFS and OS than those with low CONUT (χ^2^ = 1.844, *p* = 0.175, and χ^2^ = 5.222, *p* = 0.023, respectively; Figures 5E, F). Moreover, the 1- and 3-year survival rate for PFS and OS in the low CONUT group were 67.6% (95% CI: 53.0%–87.0%), 40.4% (95% CI: 23.7%–69.0%); and 86.1% (95% CI: 75.5%–98.2%), 55.7% (95% CI: 40.3%–77.0%), respectively. The 1-, 3-, and 5-year survival rates for PFS and OS in the high CONUT group were 62.7% (95% CI: 48.5%–81.1%), 22.6% (95% CI: 11.6%–44.2%), 18.1% (95% CI: 8.1%–40.3%); and 70.3% (95% CI: 57.0%–86.7%), 30.2% (95% CI: 17.6%–51.6%), 15.1% (95% CI: 4.9%–46.1%), respectively.

### The correlation between Controlling Nutritional Status and Toxicity Assessment

Of all enrolled patients, we evaluated and analyzed the toxicities after they received ICIs and chemotherapy. The common toxicities after treatment were hematologic reactions (anemia, leukopenia, neutropenia, and thrombocytopenia), fatigue, myelosuppression, gastrointestinal reaction, and hand–foot syndrome. Between the two groups, there was a significant association with anemia (*p* = 0.035). Moreover, between the two groups (low CONUT vs. high CONUT), there was a significant association with anemia (*p* = 0.031) in patients who received ICIs treatment, and there was a significant association with leukopenia (*p* = 0.029) in patients who received Chemotherapy treatment, respectively. The detailed information is shown in Table 5.

**TABLE 5:** The correlation between CONUT and toxicity after treatment.

n	Level	Overall	ICIs	Chemotherapy	*p*-Value	ICIs	Low CONUT	High CONUT	*p*-Value	Chemotherapy	Low CONUT	High CONUT	*p*-Value
		146	89	57		89	44	45		57	31	26	
Anemia (%)	Grade 0	109 (74.7)	67 (75.3)	42 (73.7)	0.035	67 (75.3)	38 (86.4)	29 (64.4)	0.031	42 (73.7)	25 (80.6)	17 (65.4)	0.334
	Grade 1–2	33 (22.6)	22 (24.7)	11 (19.3)		22 (24.7)	6 (13.6)	16 (35.6)		11 (19.3)	5 (16.1)	6 (23.1)	
	Grade 3–4	4 (2.7)	0 (0.0)	4 (7.0)		0 (0.0)	0 (0.0)	0 (0.0)		4 (7.0)	1 (3.2)	3 (11.5)	
Leukopenia (%)	Grade 0	133 (91.1)	84 (94.4)	49 (86.0)	0.149	84 (94.4)	43 (97.7)	41 (91.1)	0.371	49 (86.0)	30 (96.8)	19 (73.1)	0.029
	Grade 1–2	13 (8.9)	5 (5.6)	8 (14.0)		5 (5.6)	1 (2.3)	4 (8.9)		8 (14.0)	1 (3.2)	7 (26.9)	
	Grade 3–4	0 (0.0)	0 (0.0)	0 (0.0)		0 (0.0)	0 (0.0)	0 (0.0)		0 (0.0)	0 (0.0)	0 (0.0)	
Neutropenia (%)	Grade 0	138 (94.5)	87 (97.8)	51 (89.5)	0.086	87 (97.8)	43 (97.7)	44 (97.8)	1.000	51 (89.5)	28 (90.3)	23 (88.5)	0.532
	Grade 1–2	7 (4.8)	2 (2.2)	5 (8.8)		2 (2.2)	1 (2.3)	1 (2.2)		5 (8.8)	3 (9.7)	2 (7.7)	
	Grade 3–4	1 (0.7)	0 (0.0)	1 (1.8)		0 (0.0)	0 (0.0)	0 (0.0)		1 (1.8)	0 (0.0)	1 (3.8)	
Thrombocytopenia (%)	Grade 0	100 (68.5)	59 (66.3)	41 (71.9)	0.569	59 (66.3)	31 (70.5)	28 (62.2)	0.603	41 (71.9)	23 (74.2)	18 (69.2)	0.917
	Grade 1–2	31 (21.2)	19 (21.3)	12 (21.1)		19 (21.3)	9 (20.5)	10 (22.2)		12 (21.1)	6 (19.4)	6 (23.1)	
	Grade 3–4	15 (10.3)	11 (12.4)	4 (7.0)		11 (12.4)	4 (9.1)	7 (15.6)		4 (7.0)	2 (6.5)	2 (7.7)	
Fatigue (%)	No	136 (93.2)	83 (93.3)	53 (93.0)	1.000	83 (93.3)	41 (93.2)	42 (93.3)	1.000	53 (93.0)	29 (93.5)	24 (92.3)	1.000
	Yes	10 (6.8)	6 (6.7)	4 (7.0)		6 (6.7)	3 (6.8)	3 (6.7)		4 (7.0)	2 (6.5)	2 (7.7)	
Myelosuppression (%)	No	137 (93.8)	84 (94.4)	53 (93.0)	1.000	84 (94.4)	42 (95.5)	42 (93.3)	1.000	53 (93.0)	29 (93.5)	24 (92.3)	1.000
	Yes	9 (6.2)	5 (5.6)	4 (7.0)		5 (5.6)	2 (4.5)	3 (6.7)		4 (7.0)	2 (6.5)	2 (7.7)	
Gastrointestinal reaction (%)	No	118 (80.8)	70 (78.7)	48 (84.2)	0.537	70 (78.7)	35 (79.5)	35 (77.8)	1.000	48 (84.2)	26 (83.9)	22 (84.6)	1.000
	Yes	28 (19.2)	19 (21.3)	9 (15.8)		19 (21.3)	9 (20.5)	10 (22.2)		9 (15.8)	5 (16.1)	4 (15.4)	
Hand–foot syndrome (%)	No	141 (96.6)	87 (97.8)	54 (94.7)	0.609	87 (97.8)	43 (97.7)	44 (97.8)	1.000	54 (94.7)	29 (93.5)	25 (96.2)	1.000
	Yes	5 (3.4)	2 (2.2)	3 (5.3)		2 (2.2)	1 (2.3)	1 (2.2)		3 (5.3)	2 (6.5)	1 (3.8)	

### Construction of a Nomogram to Predict Progression-Free Survival and Overall Survival

**FIGURE 6:**
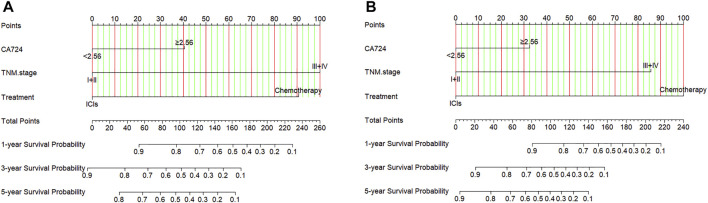
Nomogram for predicting **(A)** progression-free survival (PFS) and **(B)** overall survival (OS).

Through the results of multivariate Cox regression analysis, the CA724, TNM stage, and treatment were found to be potential prognostic factors affecting PFS and OS after ICIs or chemotherapy, and the nomogram was able to predict 1-, 3-, and 5-year PFS and OS probability using the multivariate analysis (Figures 6A, B). Moreover, the probability of 1-, 3-, and 5-year PFS was predicted with a C-index of 0.749 (95% CI: 0.659%–0.839). The probability of 1-, 3-, and 5-year OS was predicted with a C-index of 0.769 (95% CI: 0.684%–0.854).

## Discussion

In gastric cancer patients, the biomarkers of body composition, such as degree of sarcopenia, BMI, and visceral fat, have been demonstrated to be related to tumor prognosis (Imai et al., 2017; Feng et al., 2020; Loehrer et al., 2020). There is good evidence that prognosis of cancer is not only associated with tumor indicators but also patients’ condition, systemic inflammation, and nutritional status (Diakos et al., 2014; Vidra et al., 2016; Garla et al., 2018). Although surgery is the main treatment for gastric cancer, a considerable number of patients will relapse after radical resection. At present, as a result of tumor comprehension or heterogeneity, the patients may have different prognosis and vary greatly, even for the same TNM stage *via* the AJCC TNM staging system. Therefore, developing a more accurate prognostic risk stratification system to stratify patients and help guide the individualized choice of different treatments is needed.

This study was the first to assess the associations between CONUT, clinicopathological factors, and survival, and evaluate the prognostic power of CONUT in patients with gastric cancer who received ICIs treatment or chemotherapy. The results showed that the CONUT was correlated with age, pathology, and ALB, PALB, and Hb. According to the nutritional status assessment for ALB, TL, and CONUT score, patients with high ALB score, high TL score, and high CONUT score had worse survival with PFS and OS, especially with the ALB score and CONUT score. Moreover, we also analyzed the survival difference between CONUT and ICIs/Chemotherapy. It revealed that a high CONUT score was significantly associated with a poor PFS and OS, and chemotherapy in particular. The multivariate analysis showed that CA724, TNM stage, and treatment were the independent prognostic factors for PFS and OS. However, the CONUT was not the independent prognostic factor for PFS and OS in the present study. Taking into consideration the retrospective nature of this study, multiple factors might influence the multivariate analysis results, such as the patient type. In contrast, the difference in PFS and OS between the low CONUT group and the high CONUT group is more convincing. In the subgroup analysis, patients with operation treatment have better survival than those without surgery, and the patients with a high CONUT score were significantly associated with a poor PFS and OS in the Surgery subgroup. Moreover, in the subgroup analysis for PD-1/PD-L1 expression status, the patients with a high CONUT score were associated with shorter PFS and OS in patients with PD-1/PD-L1 positive expression. Furthermore, we analyzed the toxicity between ICIs and chemotherapy, and there were no significant differences among these toxic side effects, except in anemia. In the subgroup analysis, there was significant association with anemia in the ICIs group, and leukopenia in the chemotherapy group. Simultaneously, we also conducted a nomogram to predict 1-, 3-, and 5-year PFS and OS probability using the multivariate analysis results, including three independent factors, CA724, TNM stage, and treatment. The 1-, 3-, and 5-year PFS and OS survival rates in the low CONUT group were higher than those in the high CONUT group, respectively.

CONUT is a nutritional evaluation score for evaluating immune-nutritional status, and this score is an efficient tool for continuous control of undernutrition in hospitalized patients (Chen et al., 2020), and it is derived from three parameters: 1) total lymphocyte count (an indicator of loss of immune defenses caused by malnutrition), 2) serum albumin (an indicator of protein reserve), and 3) total cholesterol level (a caloric depletion parameter), which are extracted easily from a blood examination (Takagi et al., 2019). Each component of CONUT score is considered to play an important role in the occurrence, development, and progression of different cancers. Using the CONUT score to explain the above three biomarkers may provide a more accurate and comprehensive immune and nutritional status index for clinicians. In the study of Zhu X, a high CONUT was significantly related to older age, advanced TNM stage, higher Ki-67, and pathological subtype, and patients with high CONUT levels before operation should be given more observation and constant follow-up after surgery (Zhu et al., 2021). A systematic review and meta-analysis showed that the CONUT score was related to postoperative complication rate and mortality, and long-term prognosis after gastrectomy (Takagi et al., 2019). Moreover, a high CONUT score was also significantly associated with clinicopathological parameters including TNM stage and positive microvascular invasion (MVI) (Takagi et al., 2019). Another study by Ryo S indicated that the CONUT was an independent prognostic factor of OS by multivariable analysis in stage II or III gastric cancer, and the CONUT score reflected the OS for patients who underwent postoperative adjuvant chemotherapy more significantly than for those who received surgery alone (Ryo et al., 2019).

A large number of studies have indicated that the PD-1/PD-L1 pathway plays a critical role in the interaction between tumor cells and cells responsible for immune response (Zatloukalová et al., 2016). Blocking antibodies against PD-1/PD-L1 can lead to local control and persistent response in cancer patients with ineffective standard treatment (de Streel et al., 2020). Previous studies have indicated that PD-1/PD-L1 protein expression was related to the prognosis of patients in different malignant tumors (Isaacsson Velho and Antonarakis, 2018; Sui et al., 2018; Liu et al., 2020). However, the relationship between the nutritional status and PD-1/PD-L1 protein expression is still controversial. In this study, the subgroup analysis showed that the patients with a high CONUT score had worse survival than those with a low CONUT score in the PD-1/PD-L1 only positive expression. Moreover, the toxic side effects in patients with ICIs treatment were not significantly increased. A nomogram has been widely used for the purpose of predicting the prognosis of patients with various types of tumors. In this study, we constructed a nomogram in accordance with the prognostic factors performed by multivariate Cox proportional hazards regression models to predict survival outcomes in gastric cancer patients after ICIs treatment or chemotherapy. The three prognostic factors included in the nomogram for PFS and OS were CA724, TNM stage, and treatment. The C-index demonstrated that the nomogram for PFS and OS developed in this study have well discrimination. Furthermore, CA724 is the prognostic factor in this study by multivariate analyses, and the patients with a high CA724 value were significantly associated with a poor PFS and OS. Moreover, patients with a high CONUT had shorter PFS and OS than those with low CONUT, especially in patients with high CA724 value. Tong Y and others also found that CA724 was an independent factor for prognosis and could be used to TNM stage in locally advanced gastric cancer patients who received neoadjuvant chemotherapy and curative resection (Tong et al., 2021).

There are several plausible mechanisms to evaluate the relationship between CONUT and survival prognosis of gastric cancer. ALB is one of the most common parameters in evaluating immunological and nutritional status, and the ALB reduction is considered to be associated with systemic inflammation affecting hepatocyte catabolism and anabolism (Carr and Guerra, 2017; Fernández et al., 2019; Yoshida et al., 2020). Low serum albumin levels may also reflect poor hepatic functional reserve, influence the patient’s tolerance to operation, and result in worse prognosis (Wu et al., 2019). TL is an important parameter of immune condition and plays an important role in antitumor immunity by inducing cytotoxicity and inhibiting tumor cell growth, migration, and invasion (Sarvaria et al., 2017). The change in lymphocyte counts reflects the steady-state relationship in tumor resistance, development, and progression (Zhou et al., 2016; Kareva, 2017). T-CHOL is considered as an indicator of patients’ caloric reserve, thought to be related to tumor load and nutritional status, and also affecting the killing effect of immune cells on cancer cells and accordingly affecting the fluidity of the cell membrane (Yassine et al., 2009; Fallaize et al., 2018; Dong et al., 2021). Hence, the CONUT score based on these biomarkers is a comprehensive indicator of immune response, nutritional status, and systemic inflammatory response.

Despite our findings, there were still some limitations of the present study. First, it was a retrospective study; patients were obtained from just one institution, with limited sample size and potential selection bias. Considering the heterogeneity of the population, more samples from multiple centers should be included. Second, the potential factors influencing preoperative immune-nutritional status were not accessed, such as cancer-related inflammation. Third, the evaluation and assignment of the cutoff value for the CONUT score varied among reports, and the optimal cutoff value remains unclear. Therefore, further well-designed studies are warranted to identify the predictive significance of the CONUT score for gastric cancer and validate the effectiveness of patients with ICIs treatment.

## Conclusion

As a simple and feasible nutritional assessment tool, the CONUT, as a novel immuno-nutritional biomarker, may be useful in identifying gastric cancer patients who are unlikely to benefit from ICIs treatment.

## Data Availability

The raw data supporting the conclusions of this article will be made available by the authors, without undue reservation.
