# Corrigendum: Controlling nutritional status (CONUT) predicts survival in gastric cancer patients with immune checkpoint inhibitor (PD-1/PD-L1) outcomes

**DOI:** 10.3389/fphar.2022.1079635

**Published:** 2022-11-14

**Authors:** Li Chen, Hao Sun, Ruihu Zhao, Rong Huang, Hongming Pan, Yanjiao Zuo, Lele Zhang, Yingwei Xue, Hongjiang Song, Xingrui Li

**Affiliations:** ^1^ Department of Thyroid and Breast Surgery, Tongji Hospital, Tongji Medical College of Huazhong University of Science and Technology, Wuhan, China; ^2^ Department of Gastrointestinal Surgery, Harbin Medical University Cancer Hospital, Harbin Medical University, Harbin, China

**Keywords:** gastric cancer, controlling nutritional status, immune checkpoint inhibitors, PD-1, PD-L1

In the published article, there was an error in the **Author list** and **Correspondence** section, and authors Hongjiang Song and Xingrui Li were inserted in the wrong order. The corrected **Author list** and **Correspondence** section appear below.

“Li Chen, Hao Sun, Ruihu Zhao, Rong Huang, Hongming Pan, Yanjiao Zuo, Lele Zhang, Yingwei Xue, Hongjiang Song and Xingrui Li.”

“*Correspondence: Hongjiang Song, hongjiangsong2016@163.com; Xingrui Li, lixingrui@tjh.tjmu.edu.cn.”

The authors apologize for this error and state that this does not change the scientific conclusions of the article in any way. The original article has been updated.

